# Phosphatidylglycerol Incorporates into Cardiolipin to Improve Mitochondrial Activity and Inhibits Inflammation

**DOI:** 10.1038/s41598-018-23190-z

**Published:** 2018-03-20

**Authors:** Wei-Wei Chen, Yu-Jen Chao, Wan-Hsin Chang, Jui-Fen Chan, Yuan-Hao Howard Hsu

**Affiliations:** 1 Department of Chemistry, Tunghai University, Taichung, Taiwan; 2 Life Science Research Center, Tunghai University, Taichung, Taiwan

## Abstract

Chronic inflammation and concomitant oxidative stress can induce mitochondrial dysfunction due to cardiolipin (CL) abnormalities in the mitochondrial inner membrane. To examine the responses of mitochondria to inflammation, macrophage-like RAW264.7 cells were activated by Kdo2-Lipid A (KLA) in our inflammation model, and then the mitochondrial CL profile, mitochondrial activity, and the mRNA expression of CL metabolism-related genes were examined. The results demonstrated that KLA activation caused CL desaturation and the partial loss of mitochondrial activity. KLA activation also induced the gene upregulation of cyclooxygenase *(COX)-2* and phospholipid scramblase 3, and the gene downregulation of COX-1, lipoxygenase 5, and Δ-6 desaturase. We further examined the phophatidylglycerol (PG) inhibition effects on inflammation. PG supplementation resulted in a 358-fold inhibition of COX-2 mRNA expression. PG(18:1)_2_ and PG(18:2)_2_ were incorporated into CLs to considerably alter the CL profile. The decreased CL and increased monolysocardiolipin (MLCL) quantity resulted in a reduced CL/MLCL ratio. KLA-activated macrophages responded differentially to PG(18:1)_2_ and PG(18:2)_2_ supplementation. Specifically, PG(18:1)_2_ induced less changes in the CL/MLCL ratio than did PG(18:2)_2_, which resulted in a 50% reduction in the CL/MLCL ratio. However, both PG types rescued 20–30% of the mitochondrial activity that had been affected by KLA activation.

## Introduction

External infections induce the activation of toll-like receptors (TLRs) or nucleotide-binding oligomerization domain-like receptors (NLRs) to trigger inflammation^1^. Multiple enzyme types, such as phospholipases and cyclooxygenases (COXs), can be activated in macrophages to produce proinflammatory prostaglandins, followed by conversion to anti-inflammatory lipoxins^2^. Some studies have suggested that unresolved antigens may induce autoimmune disease and chronic inflammation^3,4^; in turn, chronic inflammation and concomitant oxidative stress damage mitochondrial DNA and functions, which promote aging^5^.

Mitochondria are the main source of mitochondrial reactive oxygen species (mtROS) production, which activate NLRP3 to produce proinflammatory factors^6^. Macrophage activation repurposes the mitochondria from adenosine triphosphate (ATP) synthesis to ROS production^7^, but mitochondrial defects can further increase oxidative stress and promote inflammation^8^. Mitophagy is a selective pathway to prevent mtROS accumulation and excessive inflammation^9^. The promotion of cellular respiration of blood vessels during the macrophage-mediated engulfment of steroids increases oxidative stress, which leads to cardiolipin (CL) oxidation and mitochondrial defects^10^. The reduction of oxidative stress in the mitochondria alleviates atherosclerosis and chronic inflammation^11^.

CLs are negatively charged phospholipids that are specifically localized on the mitochondria and account for 15–20% of mitochondrial lipids. Because of their unique structure with four acyl chains, CLs are responsible for the curvature formation of the mitochondrial inner membrane^12^. In addition, CLs are critical in the stabilization of protein complexes on the mitochondrial inner membrane, such as electron transfer chain (ETC)I, III, and IV, as well as ATP synthase (which ensures ETC efficiency)^13^. CLs are prone to oxidization because of their location in the mitochondria, leading to reduced ATP production^14–16^. A lack of CL remodeling and excessive CL oxidation reduces CL symmetry, which can be crucial in certain mammalian organs^17,18^. Oxidized CLs can reduce their affinity toward cytochrome *c*, and result in cytochrome *c* release into the cytosol and subsequent apoptotic pathway activation^19–23^.

CL synthase combines phosphatidylglycerol (PG) and cytidine diphosphate diacylglycerol (CDP-DAG) to synthesize CLs in eukaryotic mitochondria. These nascent CLs are further remodeled with the acyl chains from phosphatidylcholine or phosphatidylethanolamine to produce mature CLs through tafazzin (TAZ)^24^. CL remodeling alters the CL profile depending on the availability of fatty acyl chains in the environment, which can be manipulated by nutrient deprivation and lipid supplementation^25,26^. TAZ gene mutations on the X chromosome also cause mitochondrial abnormalities in patients with Barth syndrome^27^. Phospholipase (PL) A_2_ is a CL remodeling and degradation enzyme that can hydrolyze fatty acyl chains to form monolysocardiolipins (MLCLs) or dilysocardiolipins (DLCLs)^28^. According to our review of the relevant literature, eicosanoid production has never been reported to be associated with CL degradation in the mitochondria.

PG was discovered in *Scenedesmus* and contains a glycerol backbone-linked glycerol headgroup and two fatty acyl chains^29^. PG is distributed in the animal cell mitochondria as a precursor for CL synthesis^30^, and when located in lung surfactant, is critical for innate immunity regulation^31–33^. PG competes with lipopolysaccharides to disrupt the activation of the TLR pathway, thus inhibiting the formation of downstream inflammatory molecules^34,35^. However, PG-induced anti-inflammatory properties in the mitochondria have not been thoroughly studied. Therefore, we supplemented RAW264.7 cells with PG(18:1)_2_ and PG(18:2)_2_ to evaluate the effects of PG on CL synthesis and remodeling, mitochondrial functions, and the mRNA expression of CL metabolism-related genes after Kdo2-Lipid A (KLA)-induced macrophage activation.

## Results

### Mass spectrometry analysis of CLs and MLCLs in RAW264.7 cells

**Figure 1:**
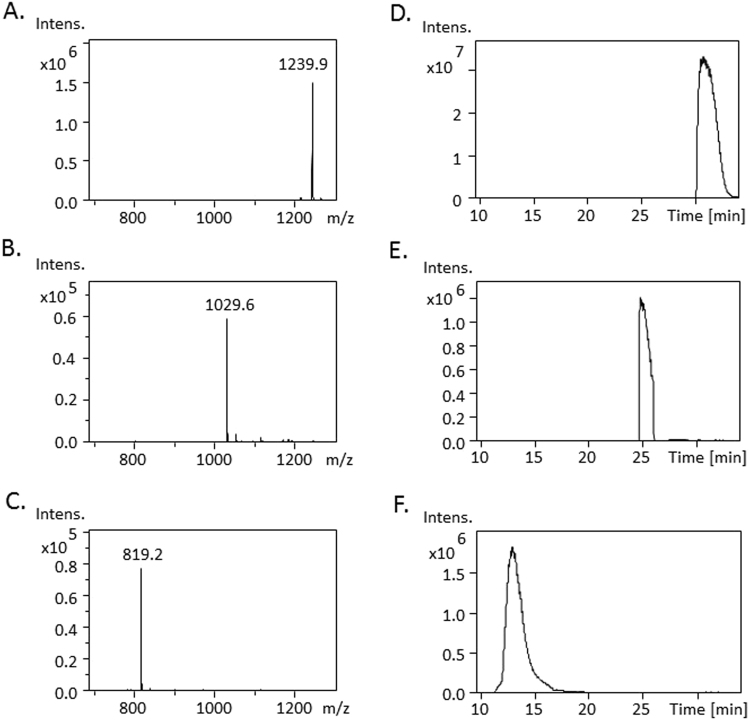
The mass spectrum and chromatography of CL, MLCL and DLCL. The CL(14:0)_4_ was hydrolyzed by sPLA_2_ to produce the monolyso-CL CL(14:0)_3_ and dilyso-CL CL(14:0)_2_, and then analyzed by a mass spectrometer with a reverse phase chromatography. The mass spectrum of (**A**) CL (**B**) MLCL (**C**) DLCL and the chromatography of (**D**) CL (**E**) MLCL **(F**) DLCL are shown.

To quantify the concentration of CLs, MLCLs, and DLCLs in a complex biological sample, we established a liquid chromatography–mass spectrometry (MS) detection method. CL(14:0)_4_ was partially hydrolyzed by secretory PLA_2_ to produce MLCLs and DLCLs, followed by a MS analysis with reverse phase chromatography (Fig. 1). The accurate retention times of these CL molecules were verified. CL(14:0)_4_ with a mass of m/z 1239.9 was eluted at 30 min, whereas the retention times of MLCL(14:0)_3_ (m/z 1029.6) and DLCL(14:0)_2_ (m/z 819.2) were 25 and 11 min, respectively. Fatty acyl hydrolysis increased the polarity of CL metabolites; therefore, MLCL and DLCL traveled faster in the C18 column.

**Figure 2:**
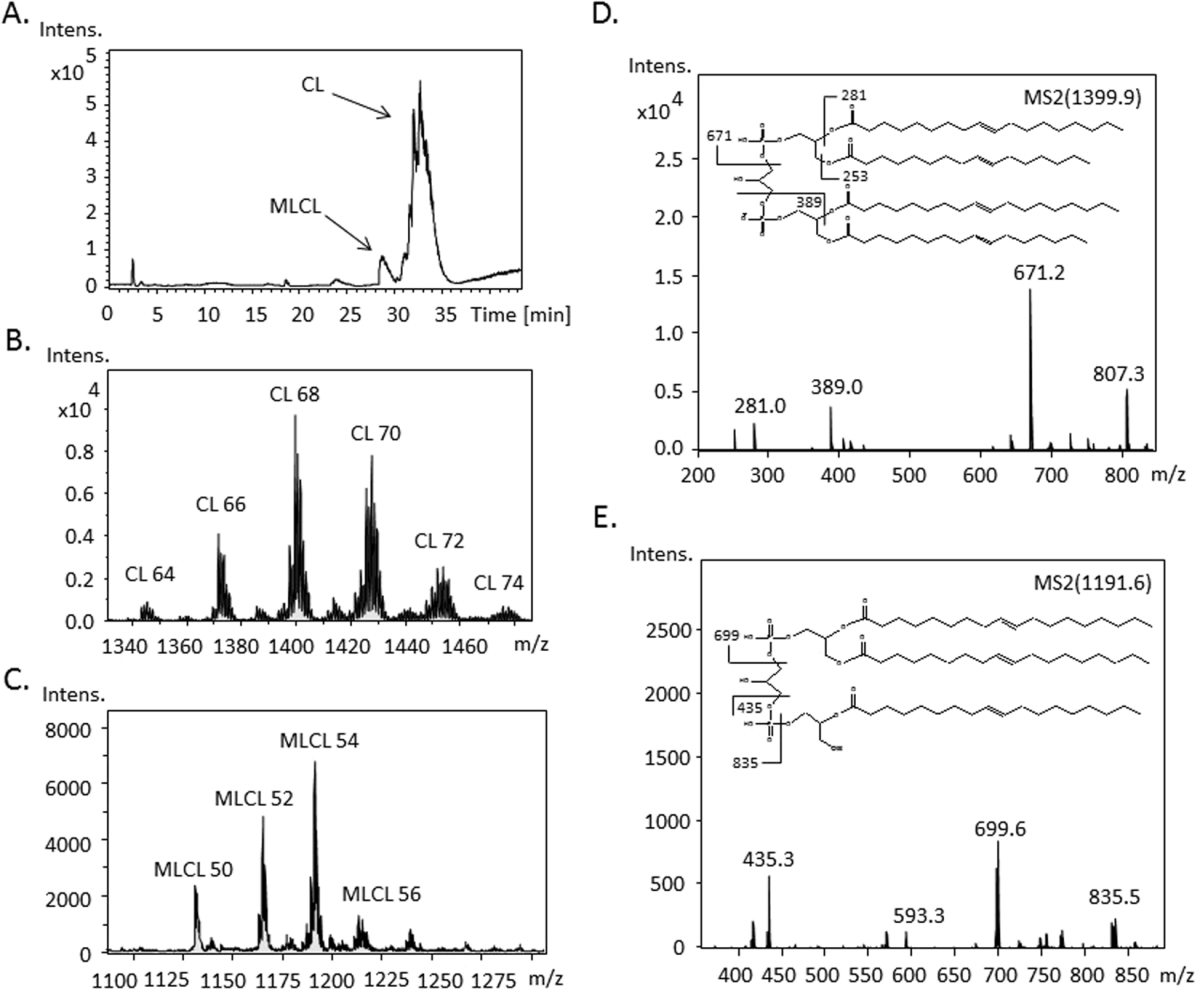
Identification of CL and MLCL in RAW264.7 cells by tandem mass spectrometry. (**A**) CL and MLCL extracted from RAW264.7 cells were separated on reverse phase chromatography. The mass spectrum of (**B**) CL and (**C**) MLCL showed in groups with different acyl chain length indicated as a number following CL and MLCL. Examples of the MS/MS fragmentation and identification of (**D**) CL and (**E**) MLCL species are shown.

CLs and MLCLs are major phospholipids of the inner mitochondrial membrane. To access the integrity of the mitochondrial membrane structure, we identified and quantified the concentration of CLs and MLCLs in RAW264.7 cells, but DLCL was too scarce to be detected. The chromatographic analysis of CLs and MLCLs in the lipid extract from RAW264.7 cells demonstrated delayed retention times compared with that of the internal CL standard (Fig. 2A); longer chain length of the extracted CLs were determined to attribute to this delay. In addition, the hydrolysis of one fatty acyl chain on CL to form MLCL slightly shifted the retention time due to increased polarity. The species of CLs and MLCLs with the same acyl chain length were categorized in groups (Fig. 2B,C). CLs had six major groups (CL64, CL66, CL68, CL70, CL72, and CL74) and MLCLs were divided in four major groups (MLCL50, MLCL52, MLCL54, and MLCL56). In addition, each group comprised 4–6 species with various double bonds. To accurately identify the fatty acyl chains in CLs and MLCLs, tandem MS was performed for phospholipid fragmentation (Fig. 2D,E). Because phosphoester bonds on phosphates and ester bonds on fatty acids are weak covalent bonds that can be easily broken, phosphatidic acid and fatty acids were the common product ions on the MS/MS spectrum.

We identified CL and MLCL species in RAW264.7 cell by LC-MS/MS. Based on the structure of CL and its charge, total lipid extraction is analyzed by reverse phase chromatography and mass spectrometry in negative mode. The methodology for CL analysis and identification are described in previous studies from our laboratory^25,26^. In our measurement, Raw 264.7 cell contains 55 CL species with 42 types of molecular weights, and each species are identified and shown in Table 1. For MLCL analysis, the signal of extracted ion chromatogram of MLCL standard (C14:0)_3_ appeared by around 24 min. The retention time of MLCL in samples, which contain more long-chain MLCL, was eluted around 26–30 min. The results showed that MLCL species shows five clusters in m/z range of 1100–1300 and 33 MLCL species with 16 molecular weights (Table 2). The identification of MLCL was carried out by secondary fragmentation. As an example, in MLCL species (C18:1)_3_, the fragments are 435, 699, and 835 m/z, which contains one acyl chain plus a phosphate group, two acyl chains plus a phosphate group, and two acyl chains with a glycerol backbone and two phosphate groups.

**Table 1:** CL identification in Raw 264.7 cell.

Species	m/z	Formula	Species	m/z	Formula
C56:0	1239.7	(14:0)(14:0)/(14:0)(14:0)	**C72:8**	1447.8	(18:2) (18:2)/(18:2)(18:2) (18:2) (20:3)/ (16:1)(18:2)
C64:2	1347.9	(16:0)(16:0)/(16:1)(16:1)	**C72:9**	1446.3	(16:1) (18:1)/(22:6) (16:1)
C64:3	1345.9	(18:1)(16:1)/(14:0)(16:1)	**C74:6**	1480.1	(20:3) (18:1)/(18:1) (18:1)
C64:4	1344.8	(16:1)(16:1)/(16:1)(16:1)	**C74:7**	1477.8	(18:2) (18:1)/(20:3) (18:1)* (18:1) (18:1)/(20:3) (18:2)
C66:1	1377.8	(16:0)(18:0)/(16:1)(16:0)			
C66:2	1375.9	(16:0)(18:1)/(16:0)(16:1)	**C74:8**	1475.8	(20:3)(16:0)/(20:4)(18:1)* (20:3)(20:4)/(16:0)(18:1) (18:1)(16:1)/(20:3)(20:3)
C66:3	1373.9	(16:0)(16:1)/(16:0)(18:2)			
C66:4	1371.9	(18:1)(16:1)/(16:1)(16:1)			
C66:5	1370.7	(16:1)(16:1)/(16:1)(18:2)	**C74:9**	1473.8	(20:3) (18:2)/(18:2) (18:2)
C68:2	1403.8	(16:0)(18:1)/(16:0)(18:1)	**C76:8**	1503.8	(22:4)(18:1)/(18:2)(18:1)
C68:3	1402.0	(16:1)(18:1)/(18:1)(16:0)	**C76:9**	1500.7	(20:3)(18:1)/(20:4)(18:1)* (18:1)(18:2)/(20:3)(20:3)
C68:4	1399.9	(16:1)(18:1)/(16:1)(18:1)			
C68:5	1397.8	(16:1)(18:2)/(18:1)(16:1)	**C76:10**	1499.2	(20:4)(18:1)/(20:4)(18:1)* (18:1)(18:1)/(20:4)(20:4)
C68:6	1395.7	(18:2)(16:1)/(18:2)(16:1)			
C70:2	1432.3	(18:1)(18:0)/(18:1)(16:0)	**C76:11**	1497.7	(20:4)(18:2)/(20:4)(18:1)* (20:3)(20:4)/(18:2)(18:2) (18:1)(18:2)/(20:4)(20:4)
C70:3	1429.8	(18:1)(18:1)/(16:0)(18:1)			
C70:4	1427.9	(18:1)(18:1)/(18:1)(16:1)* (16:0)(18:1)/(18:1)(18:2)			
			**C76:12**	1496.3	(20:4)(20:4)/(18:3)(18:1)
C70:5	1425.8	(18:1)(18:2)/(16:1)(18:1)* (18:1)(18:3)/(18:1)(16:0)	**C76:13**	1492.6	(20:3)(20:4)/(20:4)(18:2)* (20:4)(20:4)/(20:4)(18:1) (20:4)(20:4)/(20:3)(18:2)
C70:6	1424.6	(16:1)(18:2)/(18:1)(18:2)			
C70:7	1420.8	(16:1)(18:2)/(16:1)(20:3)	**C78:9**	1528.4	(22:3)(18:1)/(20:4)(18:1)* (20:2)(18:1)/(20:4)(20:2)
C72:3	1457.8	(18:1)(18:1)/(18:1)(18:0)	**C78:10**	1527.6	(20:4)(18:0)/(20:4)(20:2)
C72:4	1455.8	(18:1) (18:1)/(18:1) (18:1)	**C78:11**	1524.7	(20:4)(18:1)/(20:3)(20:3)
C72:5	1453.8	(18:1) (18:2)/(18:1) (18:1)	**C78:12**	1523.5	(20:4)(18:1)/(22:5)(18:2)
C72:6	1451.8	(18:1) (18:2)/(18:1) (18:2)	**C78:13**	1521.8	(18:1)(20:4)/(20:4)(20:4)
C72:7	1449.8	(18:1) (18:2)/(18:2) (18:2)* (16:1) (18:1)/(18:2) (20:3)	Bold: identified species *main species

**Table 2:** MLCL identification in RAW264.7 cell.

Species	m/z	Formula	Species	m/z	Formula
**C50:3**	1135.80	(34:2)/(16:1) (32:0)/(18:1)	**C56:7**	1211.81	(36:3)/(20:4) (38:6)/(18:1) (38:4)/(18:3)
**C50:2**	1137.73	(16:0)(16:1)/(18:1) (34:2)/(16:0)			
			**C56:6**	1213.63	(36:2)/(20:4) (38:5)(18:1) (38:6)/(18:1)
**C52:3**	1164.11	(34:2)/(18:1) (36:2/(16:1)			
**C52:2**	1165.98	(34:1)/(18:1) (36:2)/(16:0)	**C56:5**	1215.83	(38:4)/(18:1) (36:1)/(20:4)
**C53:4**	1175.94	(36:2)/(17:2) (36:3)/(17:1) (35:3)/(18:1)	**C56:4**	1217.83	(38:3)/(18:1) (38:4)/(18:0)
			**C58:8**	1238.01	(40:8)/(18:0) (40:7)/(18:1) (36:2)/(22:6)
**C53:4**	1176.48	(35:2)/(18:2)			
**C53:3**	1177.12	(36:2)/(17:1) (35:2)/(18:1)			
			**C58:5**	1241.67	(36:2)/(22:4) (40:6)/(18:0) (36:1/(22:5) (40:4)/(18:2)
**C54:5**	1187.78	(36:4)/(18:1) (36:3)/(18:2) (34:1/(20:4)			
**C54:4**	1189.78	(36:3)/(18:1) (36:2)/(18:2) (34:1)/(20:3)	Bold: identified species. *:Main species. *a*: predicted formula		
**C54:3**	1191.62	(36:2)/(18:1)(36:3)/(18:0)			

### Incorporation of PG(18:1)_2_ into CLs and phospholipids

**Figure 3:**
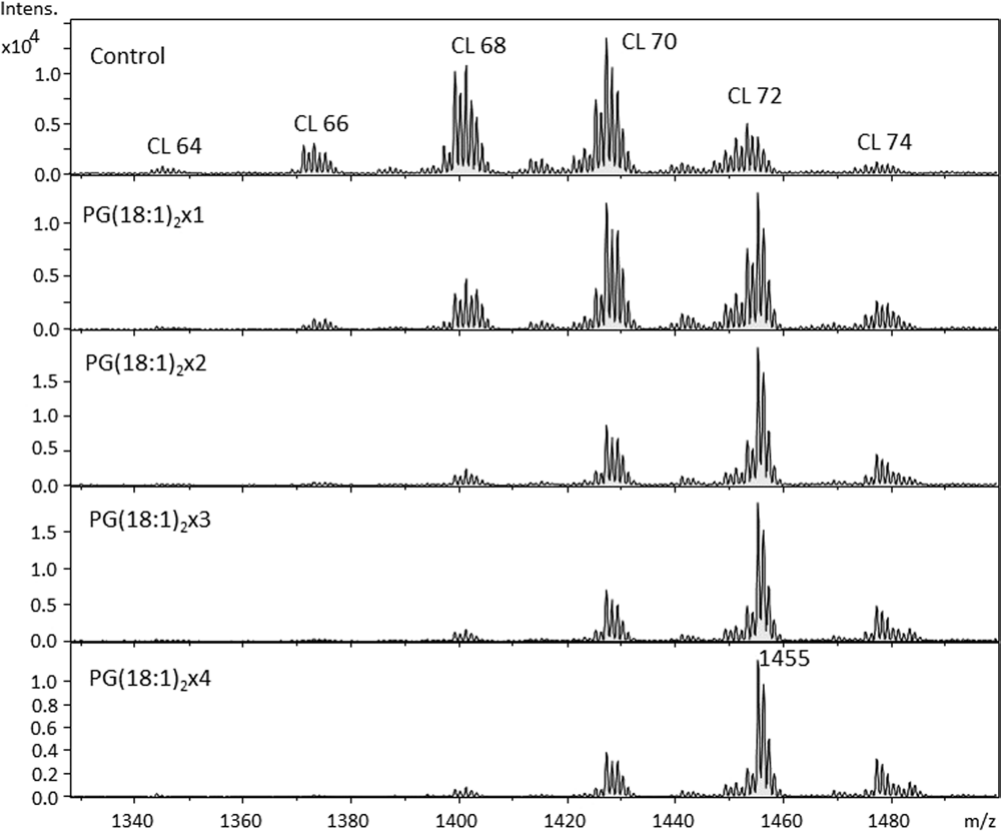
The mass spectrum of the CL in RAW264.7 after PG(18:1)_2_ supplementation. (**A**) The CL pattern of RAW264.7 was analyzed by LC-MS. RAW264.7 cells are further supplemented with 1 to 4 times of 50 μM PG in a 12-hour interval. The addition was at time (**B**) 0, (**C**) 12, (**D**) 24, (**E**) 36 hours. All cells were harvested at 48 hours.

CLs are biologically synthesized from PG and CDP-DAG by CL synthase in the mitochondria of mammalian cells. We attempted to manipulate the CL contents in RAW264.7 cells by supplementation with PG and CDP-DAG. The optimal concentrations of PG and CDP-DAG were determined through MTT assay (Supplemental Fig. S1). Cells were supplemented with 50 μM of PG and CDP-DAG, and the medium was supplemented with 50 μM PG(18:1)_2_ four times every 12 hours period. The CL patterns in PG(18:1)_2_-supplemented cells were then analyzed through MS (Fig. 3). The chain length of CL increased drastically, and the diversity of the CL groups decreased. After the second supplementation, only three major CL groups were detected (CL70, CL72, and CL74), with CL72 being predominant. Notably, the mass peak in CL72 at m/z 1455.4 was for the symmetrical CL(18:1)_4_, which indicates the incorporation of the 18:1 fatty acyl chain into CLs. After the second supplementation, CL(18:1)_4_ was the most abundant CL species in RAW264.7 cells, which suggests the efficient incorporation of the 18:1 fatty acyl chain from PG to CL in the first and second supplementation. PG(18:1)_2_ supplementation remarkably increased the percentage of symmetrical CLs. Furthermore, we evaluated the effects of PG(18:1)_2_ supplementation in fresh media to RAW264.7 cells during cell passage (Supplemental Fig. S2). Although the initial supplementation significantly altered the CL profile, the effects were minimal, despite the 8-day maintenance of the experiments. Therefore, these results reveal that two-time PG supplementation is the optimal supplementation method for CL remodeling; it was the standard protocol used in the following experiments for the present study.

**Figure 4:**
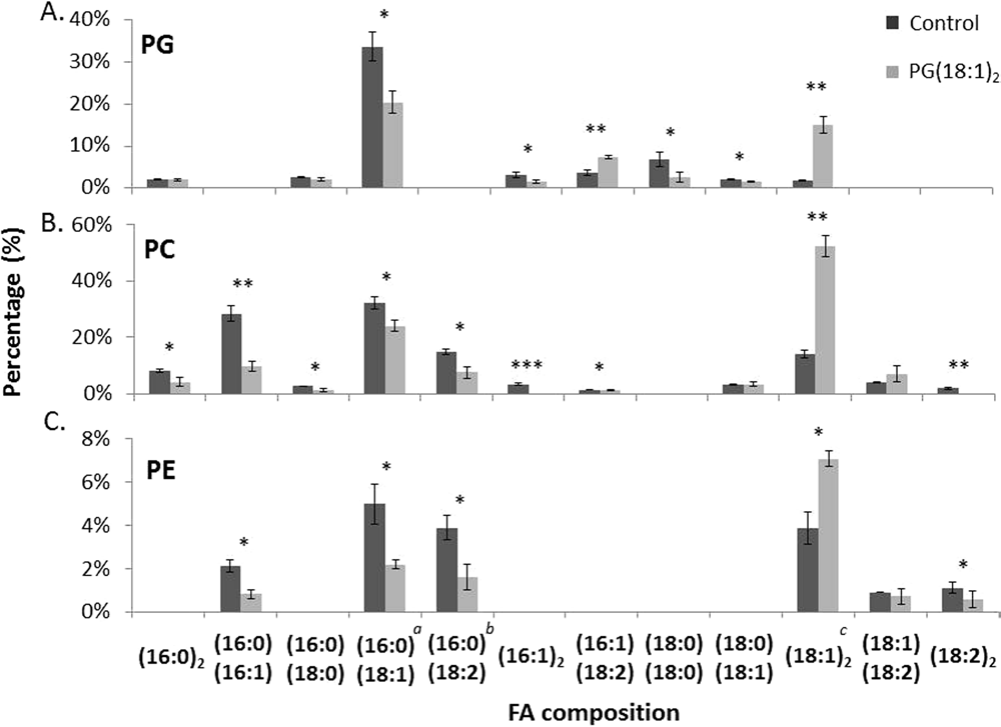
Mitochondrial phospholipid changes upon PG supplementation. RAW264.7 cells were supplemented with 50 μM of PG(18:1)_2_ twice. Mitochondria were purified and the phospholipids were then extracted by Bligh-Dyer’s method. The (**A**) PG, (**B**) PC and (**C**) PS in the sample were analyzed by LC-MS/MS. Minor species, *a*: (16:1)(18:0), *b*: (16:1)(18:1) and *c*: (18:0)(18:2) were also detected with the dominant species. The experiments were performed in triplicate and statistically analyzed by t-test (*p < 0.05, **p < 0.01, ***p < 0.001).

We further examined the two-time PG supplementation effects on phosphatidylglycerol (PG), phosphatidylcholine (PC) and phosphatidylethanolamine (PE) (Fig. 4). After PG(18:1)_2_ supplementation, the concentration of PG(18:1)_2_ in mitochondria increased 9.6 folds, indicating the supplemented PG(18:1)_2_ successfully entering mitochondria. The concentrations of PC(18:1)_2_ and PS(18:1)_2_ increases 2.4 folds and 1.4 folds respectively. These results indicated that the imported PG(18:1)_2_ in mitochondria stimulated either the conversions among PG, PC and PE phospholipids or the remodeling of PC and PE in mitochondria.

### PG(18:1)_2_ supplementation produces a higher percentage of symmetrical CLs than does PG(18:2)_2_ supplementation

**Figure 5:**
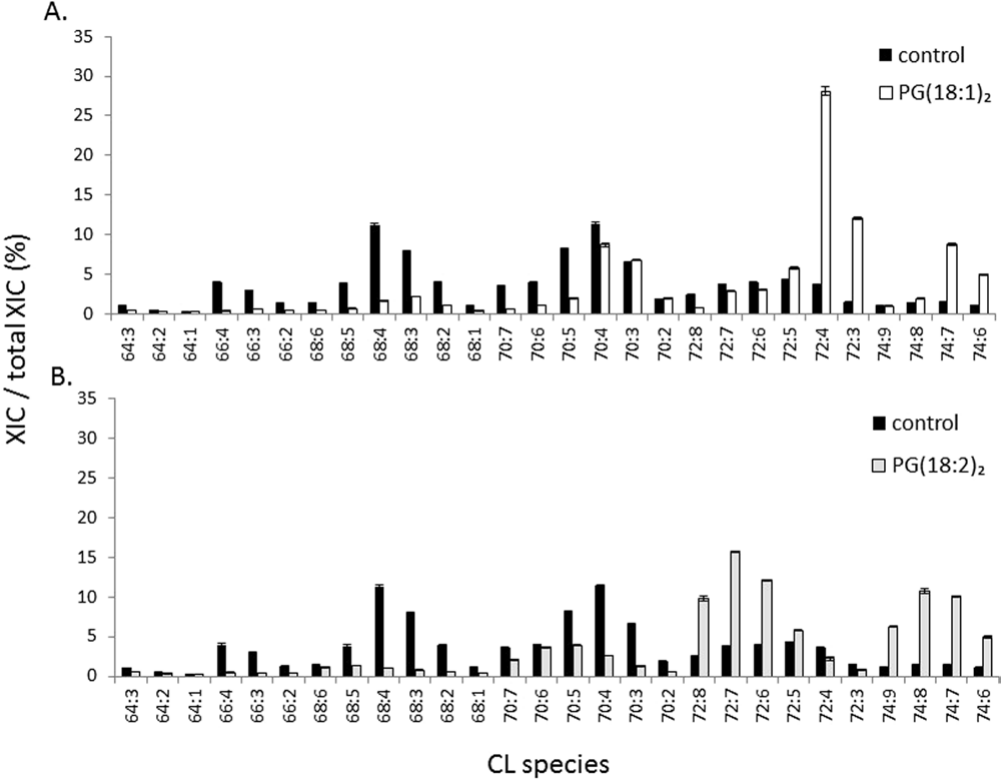
Analysis of the percentages of CL species in RAW264.7 cells after PG supplementation. RAW264.7 cells were supplemented with 50 μM of (**A**) PG(18:1)_2_ and (**B**) PG(18:2)_2_ twice at time 0 and 12 hours. The cells were harvested at 48 hours. The 28 CL species were analyzed by IonTrap mass spectrometry.

CLs are mainly composed of 18:1 and 18:2 fatty acyl chains in animal cells. CL symmetry is also critical for CL maturation^17^. As indicated in the preceding section, we supplemented RAW264.7 cells with 50 μM of PG(18:1)_2_ and PG(18:2)_2_ twice to alter CL compositions (Fig. 5). PG(18:1)_2_ supplementation increased the dominant CL chain length to CL72. Specifically, CL(72:3) and CL(72:4) increased from 1.4% to 12.1% and from 3.6% to 28.1%, respectively, which were the species with the highest increase percentage. By contrast, CL66 and CL68 were the groups with decreasing percentages, most evident within CL(66:4) and CL(68:4) (3.6% and 9.56% decrease, respectively). Additionally, CL70, C72, and C74 showed a significant reduction in CL saturation.

Following PG(18:2)_2_ supplementation, the shift of CL68 and CL70 to CL72 and CL74, respectively, was significant. In addition to the increase in chain length, CL saturation decreased drastically. The species with more than four double bonds increased in percentage, which strongly indicates the incorporation of the 18:2 fatty acyl chain to replace the fatty acids with 0–1 double bonds. CL(72:6), CL(72:7), and CL(72:8) in CL72, and CL(74:7), CL(74:8), and CL(74:9) in CL74 showed a significant increase in percentage. Conversely, CL(68:4) decreased by 10.2%, which is the most remodeled species.

In short, the CL70, CL72, and CL74 groups showed a high variation in CL species after PG(18:1)_2_ and PG(18:2)_2_ supplementation. PG(18:1)_2_ supplementation produced CLs with 2–4 double bonds, and PG(18:2)_2_ supplementation produced CLs with 5–8 double bonds. We also confirmed that the PG(18:1)_2_ and PG(18:2)_2_ supplementation increases the abundance of 18:1 and 18:2 fatty acyl chain in CL respectively.

**Figure 6:**
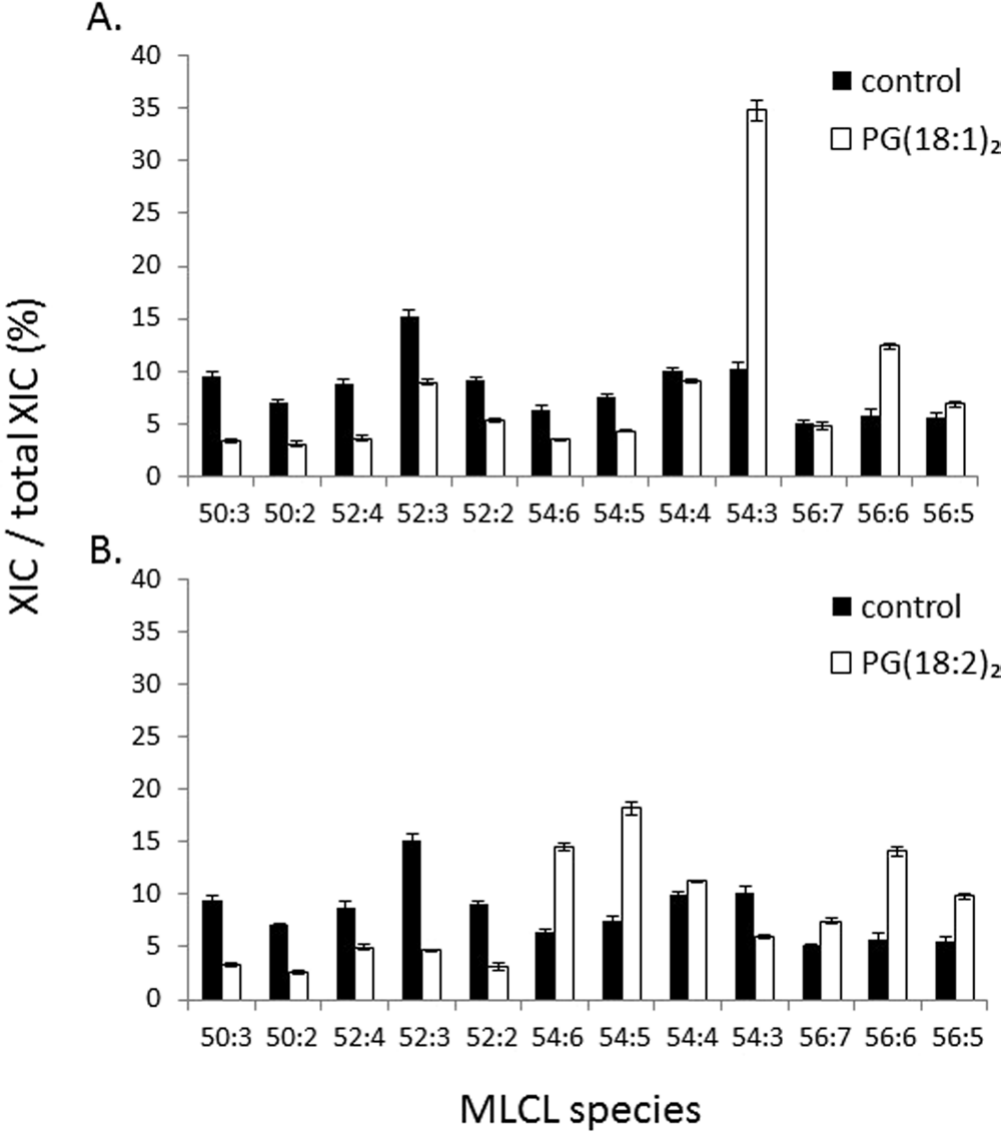
PG supplementation effects on MLCL in RAW264.7. RAW264.7 cells were supplemented with 50 μM of (**A**) PG(18:1)_2_ and (**B**) PG(18:2)_2_ at time 0 and 12 hours. The cells were harvested at 48 hours. There were 12 MLCL species analyzed by IonTrap mass spectrometry.

MLCLs are considered to be the hydrolyzed products of CLs because the biological synthesis of MLCLs has yet to be reported; nevertheless, it is suspected that the species and concentrations of MLCLs are strongly related to those of CLs. In the present study, we examined the changes in MLCLs after PG supplementation (Fig. 6). After the addition of PG(18:1)_2_, MLCL(54:3) increased drastically from 10.16% to 34.84% to become the major MLCL species. In addition, MLCL(56:6) showed a significant percentage increase of 6.6%. However, the percentages of the short chain MLCL50 and MLCL52 species decreased. PG(18:2)_2_ supplementation resulted in a significant increase of 10.6%, 8.2%, 4.3%, and 8.3% in MLCL(54:5), MLCL(54:6), MLCL(56:5), and MLCL(56:6), respectively. PG(18:1)_2_ supplementation specifically increased the percentage of MLCL(18:1)_3_, which is different from the effects of PG(18:2)_2_ supplementation that increased the percentage of multiple unsaturated species. These effects on MLCLs are in accordance with those on CLs, supporting the conceptualization that MLCLs are the hydrolysis products of CLs.

However, a 50-μM CDP-DAG supplementation of RAW264.7 cells resulted in nonsignificant changes in both CLs and MLCLs (Supplemental Fig. S3), suggesting that CDP-DAG may be unable to incorporate into CL through external supplementation.

### Effects of PG supplementation and KLA activation on CLs and MLCLs

**Figure 7:**
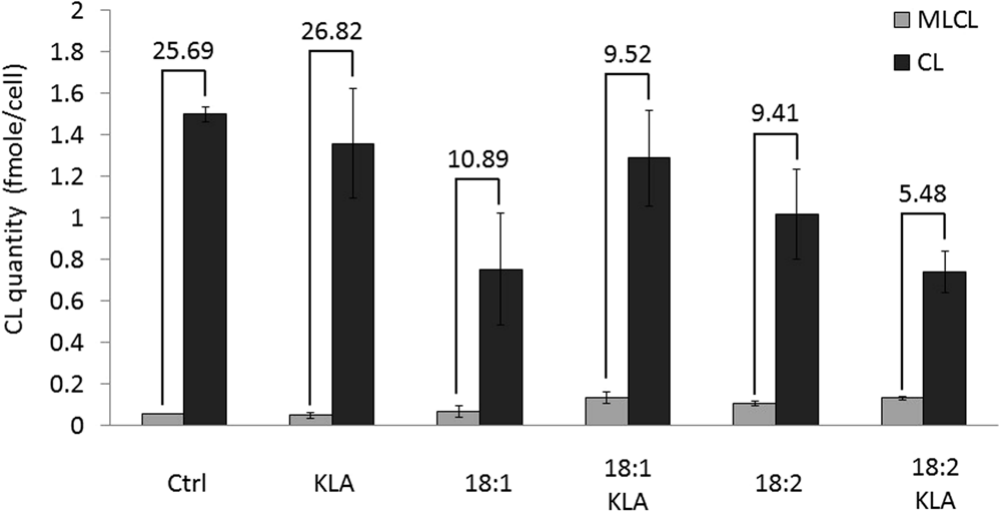
CL and MLCL changes in RAW264.7 upon PG supplementation and KLA activation. The RAW264.7 cells were primed with PG for 24 hours and then treated with KLA for another 24 hours before harvest. The quantity of CL and MLCL is based on the sum of the MS XIC of CL or MLCL to internal standard. The numbers above the bars are the value of CL/MLCL.

In the RAW264.7 inflammation model, KLA-induced TLR4 activation altered the phospholipid composition and CL saturation^36^. PG suppresses inflammation^37^, and in the present study, PG supplementation drastically changed CL compositions in RAW264.7 cells (Fig. 3); therefore, the mechanisms through which PG suppresses inflammation and affects CL compositions during macrophage activation are worth exploring. We supplemented KLA (TLR-4-specific agonist)-activated cells with PG and analyzed the quantity of CLs and MLCLs and the CL/MLCL ratio (Fig. 7). The CL and MLCL quantity in RAW264.7 cells were 1.50 and 0.06 fmole/cell, respectively, with a CL/MLCL ratio of 25.6. After KLA activation for 24 hours, the quantity of CLs and MLCLs per cell did not change significantly. After PG(18:1)_2_ supplementation for 48 hours, the CL quantity decreased to 0.75 fmole/cell, which reduced the CL/MLCL ratio to 10.8. After both PG(18:1)_2_ supplementation and KLA activation, the CL quantity was 1.29 fmole/cell, and the MLCL quanitity was 0.14 fmole/cell. Therefore, the CL/MLCL ratio did not fluctuate and was maintained at 9.5.

After PG(18:2)_2_ supplementation for 48 hours, the CL and MLCL quantities were 1.02 and 0.11 fmole/cell, respectively, with a CL/MLCL ratio of 9.4. This is similar to the quantity changes that occurred after PG(18:1)_2_ supplementation. However, KLA activation of PG(18:2)_2_-supplemented cells resulted in a drastic decrease in the CL/MLCL ratio to 5.4. Although supplementation with both PG types decreased the CL concentration and increased the MLCL concentration, PG(18:2)_2_-supplemented cells tended to increase the MLCL percentage of the total CL species after KLA activation, indicating that CL(18:2)_4_ is an unfavorable CL species after KLA activation.

**Figure 8:**
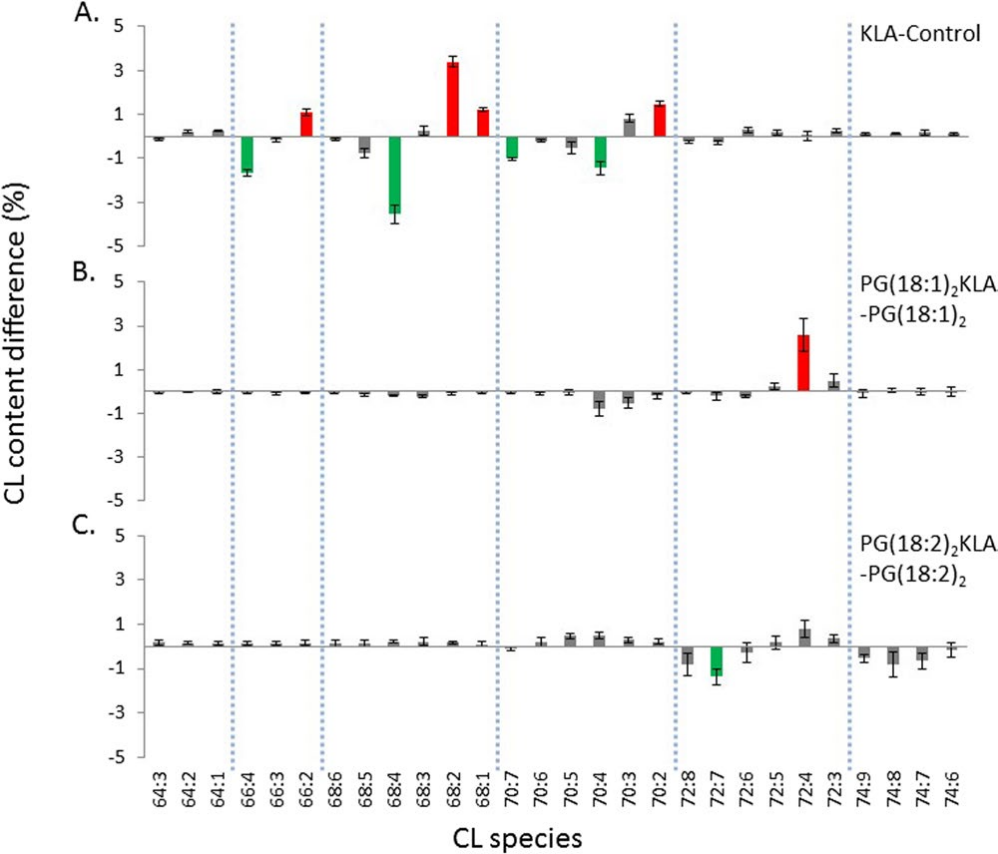
CL fluctuation in the PG supplemented cells upon KLA activation. (**A**) RAW264.7 cells, and (**B**) the PG(18:1)_2_ and (**C**) PG(18:2)_2_ supplemented cells were treated with KLA. The changes of the percentages of the CL contents in the three PG supplementation conditions upon KLA activation were shown. The percentage increases over 1% are colored in red and decreases over 1% are colored in green. The dashed straight lines are the division of groups of CL and MLCL with the same carbon numbers.

KLA triggered differential changes in the CL composition of PG-supplemented RAW264.7 cells (Fig. 8A). After the KLA activation of cells without PG supplementation, the CL species with increased percentages were as follows: CL(66:2), 1.12%; CL(68:1), 1.22%; CL(68:2), 3.40%; and CL(70:2), 1.46%. All of these species had less than two double bonds. By contrast, the CL species with decreased percentages had more than four double bonds and are listed as follows: CL(66:4), 1.65%; CL(68:4), 3.54%; CL(70:4), 1.43%; and CL(70:7), 1.03%. Because the CL species has been identified, we were able to examine the KLA effects on the saturation of CL. Upon KLA treatment, the esterified fatty acid 16:1 on CL (16:1)(18:1)/(16:1)(18:1) were saturated to 16:0 on CL (16:0)(18:1)/(16:0)(18:1) to increase the overall CL saturation. When cells were pretreated with PG(18:1)_2_ to alter CL compositions and subsequently activated by KLA (Fig. 8B), most of the CL species did not change except CL(72:4), and the symmetrical CL(18:1)_4_ remained the most abundant CL species. When cells were pretreated with less saturated PG(18:2)_2_ and KLA (Fig. 8C), CL saturation did not change drastically, except a slight 1.38% decrease in CL(72:7). The percentage of symmetrical CL(18:2)_4_ was expected to increase after PG(18:2)_2_ supplementation; however, it decreased.

### PG supplementation rescues mitochondrial activity in RAW264.7 cells after KLA activation

**Figure 9:**
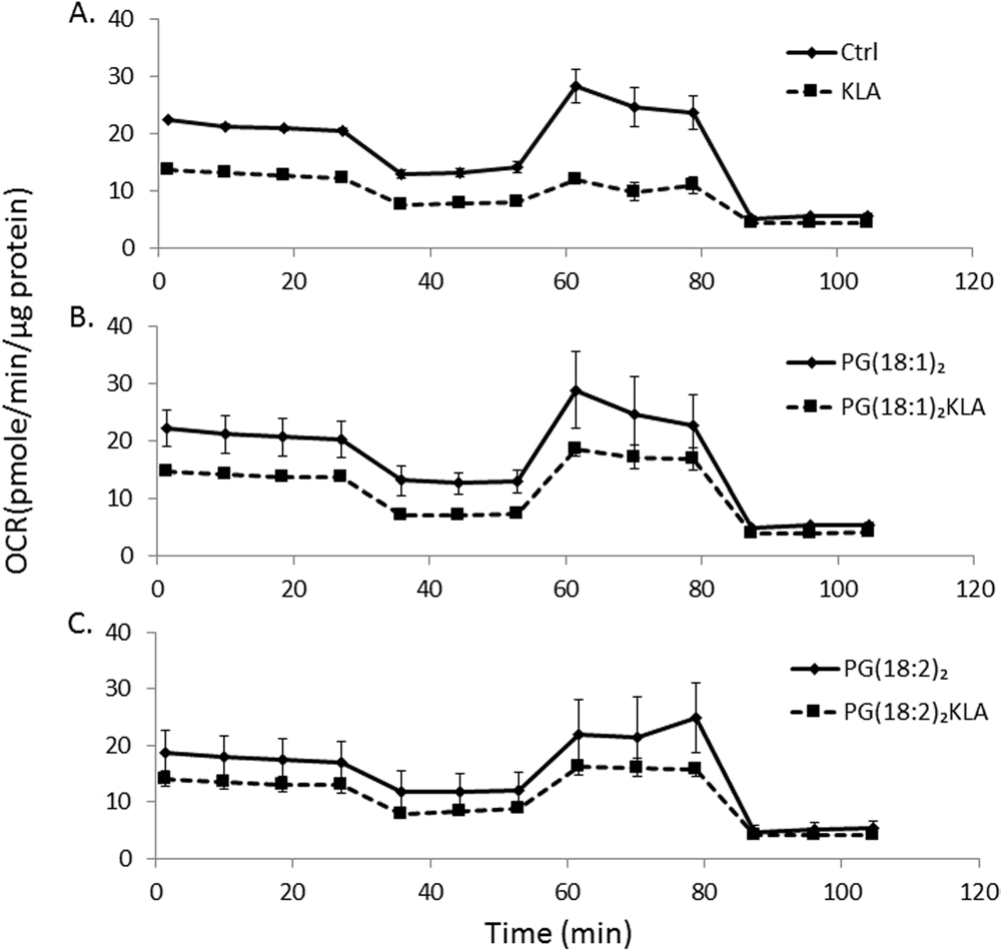
Mitochondrial activity in RAW264.7 cells. The oxygen consumption rates (OCR) analyzed by using the seahorse XF-24 extracellular flux analyzer. The OCR value in RAW264.7 cells of control and activation groups (**A**), PG(18:1)_2_ treatment and PG(18:1)_2_ treatment with activation groups (**B**), PG(18:2)_2_ treatment and PG(18:2)_2_ treatment with activation groups (**C**) are normalized with their protein contents in each groups by Bradford protein assay.

After PG supplementation and KLA treatment, the mitochondrial activity in RAW264.7 cells was examined using a Seahorse XF-24 extracellular flux analyzer (Fig. 9). The overall oxygen consumption rates (OCRs) decreased after KLA activation in the absence of PG (Fig. 9A). Specifically, the OCRs from basal respiration, ATP production, and maximal respiration were significantly reduced by 46%, 35%, and 67%, respectively.

Without KLA activation, PG(18:1)_2_-supplemented cells did not exhibit significant changes in the control group (Figs. 9A,B). However, KLA-induced activation resulted in reduced OCRs in PG(18:1)_2_-supplemented cells (Fig. 9B). For these cells, the OCRs from basal respiration, ATP production, and maximal respiration decreased by 37%, 17%, and 33%, respectively. In other words, PG(18:1)_2_ supplementation rescued the mitochondrial activity.

In PG(18:2)_2_-supplemented cells, KLA reduced the OCRs of the mitochondria in RAW264.7 cells (Fig. 9C); specifically, those from basal respiration, ATP production, and maximal respiration decreased by 27%, 13%, and 33%, respectively. After KLA activation, compared with PG(18:1)_2_-supplemented cells, PG(18:2)_2_-supplemented cells exhibited a slightly alleviated the mitochondrial activity, which is probably due to the original reduction in PG(18:2)_2_ supplementation control.

### mRNA expression of CL metabolism-related genes

**Figure 10:**
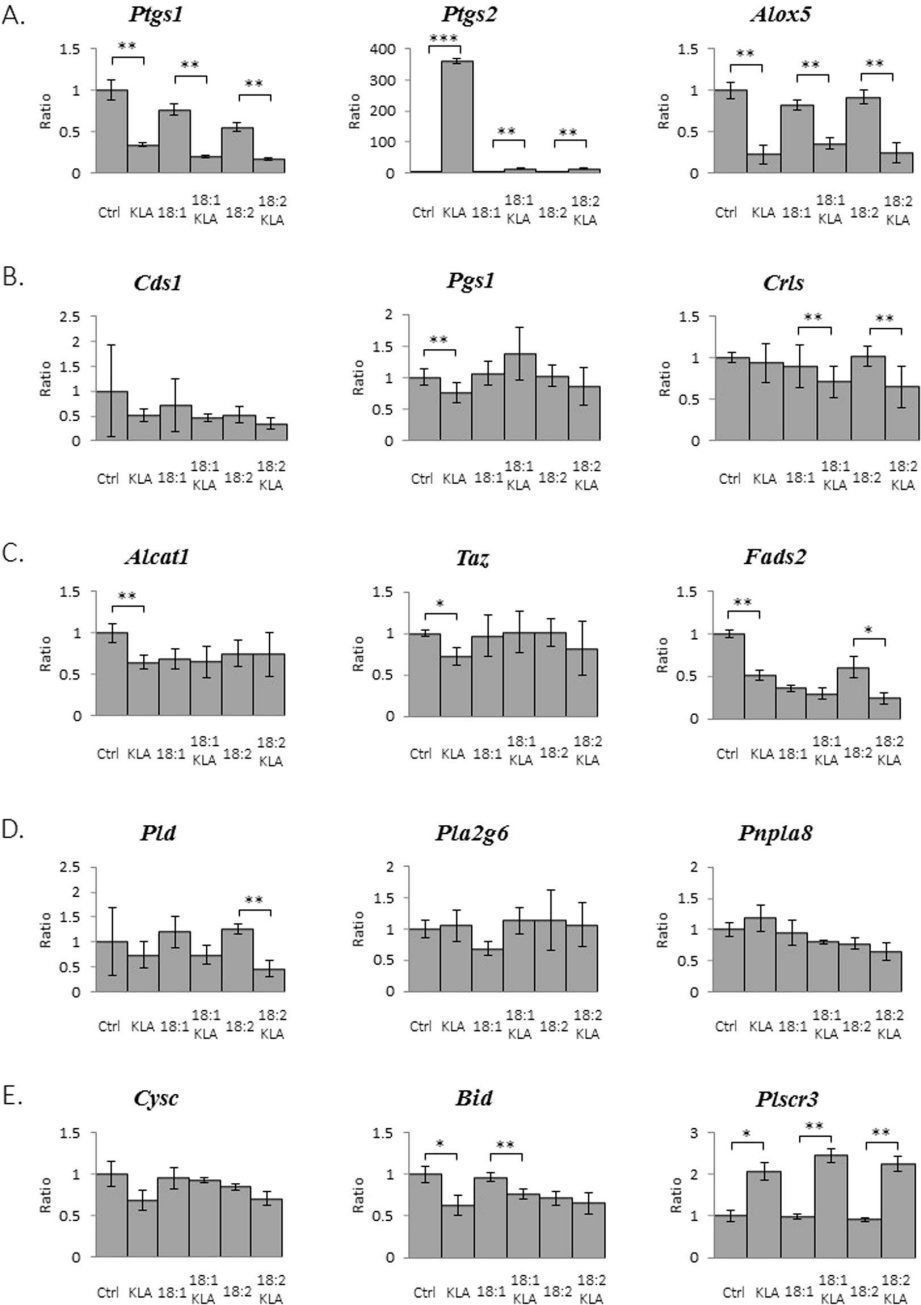
KLA activation and PG supplementation change the gene expression level in RAW264.7 cells. RAW264.7 cells treated with KLA and/or PG(18:1) or PG(18:2) were collected. The expression levels of (**A**) eicosanoid synthesis: *Ptgs1*, *Ptgs2*, *Alox5* (**B**) CL synthesis: *Cds1*, *Pgs1*, *Crls* (**C**) CL remodeling: *Alcat1*, *Taz*, *Fads2* (**D**) CL catabolism: *Pld*, *Pla2g6*, *Pnpla8* and (**E**) apoptosis: *Cysc*, *Bid*, *Plscr3* related genes were quantitated by RT-qPCR. The data were triplicated and statically analyzed by Microsoft Excel t-Test (*p < 0.05, **p < 0.01, ***p < 0.001).

To further investigate the gene regulations associated with CL and MLCL profile changes in response to KLA activation and PG supplementation, we analyzed the mRNA expression of CL-metabolism-related genes through reverse transcription quantitative real-time polymerase chain reaction (Fig. 10). Three representative genes related to inflammatory responses, namely *Ptgs1*, *Ptgs2*, and *Alox5*, were evaluated (Fig. 10A). PG(18:1)_2_ and PG(18:2)_2_ supplementation alone in RAW264.7 cells resulted in 23% and 45% reductions in the mRNA expression of *Ptgs1*, respectively; however, no apparent effects were observed on the mRNA expression of *Ptgs2* and *Alox5*. In RAW264.7 cells treated with KLA, the mRNA expression of *Ptgs1* and *Alox5* decreased by 66% and 77%, respectively, but that of *Ptgs2* increased significantly by 358 folds, indicating significant macrophage activation.

After PG supplementation, KLA-induced inflammation reduced drastically. KLA did not affect the mRNA expression of *Ptgs1* and *Alox5*, even after the supplementation of either PG(18:1)_2_ or PG(18:2)_2_, compared with cells without PG supplementation. However, both PG(18:1)_2_ and PG(18:2)_2_ drastically reduced the KLA-induced mRNA expression of *Ptgs2* by 27.5 folds.

The mRNA expression of CL synthesis-related genes did not exhibit significant changes after KLA treatment and PG supplementation (Fig. 10B). Acyl-CoA:lysocardiolipin acyltransferase-1(*Alcat1*) and *Taz* are crucial in mitochondrial CL remodeling (Fig. 10C). When RAW264.7 cells were treated with KLA, the mRNA expression of *Alcat1* and *Taz* decreased by 36% and 28% relative to the control group; however, other treatments exerted no significant effects. Furthermore, we analyzed the mRNA expression of fatty acid desaturase 2 (*Fads2*), which is associated with fatty acid desaturation, which is an important step during fatty acid elongation. The mRNA expression of *Fads2* decreased by 49% after KLA treatment, and decreased by 64% and 40% after PG(18:1)_2_ and PG(18:2)_2_ supplementation, respectively. Additionally, a 40% downregulation of *Fads2* mRNA expression was observed in cells with KLA and PG(18:2)_2_ supplementation compared with those with only PG(18:2)_2_ supplementation. This phenomenon was not observed in cells with PG(18:1)_2_ supplementation alone.

KLA treatment and PG supplementation did not significantly change the mRNA expression of CL hydrolysis-related genes (Fig. 10D). However, the mRNA expression of *Pld* decreased by 64% after KLA activation in PG(18:2)_2_-supplemented cells, and *Pla2g6* mRNA expression increased by 65% after KLA activation in PG(18:1)_2_-supplemented cells.

Cytochrome *c* (*Cycs*), BH3-interacting domain death agonist (*Bid*), and phospholipid scramblase 3 (*Plscr3*) were the genes associated with apoptosis regulation (Fig. 10E). In RAW264.7 cells treated with KLA, the mRNA expression of *Cycs* and *Bid* decreased by 32% and 37%, respectively, but that of *Plscr3* increased by 108%. PG supplementation alleviated the effects of KLA on the mRNA expression of *Cycs* and *Bid*; however, the mRNA expression of *Plscr3* was not affected or was only slightly affected by PG supplementation.

## Discussion

KLA triggered the activation of macrophage-like RAW264.7 cells and stimulated the activation of TLR4, which stimulates the activation of PLA_2_, COX-1, and COX-2 to produce prostaglandins and leukotrienes. We observed a drastic 358-fold increase in the mRNA expression of *Ptgs2* genes after KLA activation. However, the mRNA expression of *Ptgs1* and *Alox5* decreased by 66% and 77%, respectively. Thus, KLA mainly stimulated *Ptgs2*, but not *Ptgs1* and *Alox5*, to produce prostaglandins in RAW264.7 cells.

Upon macrophage activation, the total quantity of CLs and MLCLs per cell did not change; however, the species became highly saturated, most likely due to the 50% downregulation of Δ-6 desaturase mRNA expression. However, the enzymes related to CL synthesis and remodeling were either unaffected or slightly inhibited, and the changes in CL species were not attributed to the gene regulation of CL synthesis and remodeling. Furthermore, the degradation-related genes, sPLA_2_ and PLD, were not induced. The present results strongly indicate that macrophage activation inhibits the elongation of fatty acid.

The activated cells altered their CL profiles to become highly saturated CL species, which may prevent the oxidation of double bonds in activated macrophages; however, this process may also reduce mitochondrial activity. Notably, a mitochondrial activity analysis clearly demonstrated a reduction in the OCRs from basal respiration and ATP production, and particularly, a 67% reduction in the OCR from maximal respiration. In this KLA-induced activation process, the mitochondria lost more than half of its capability to provide energy to cells. Studies have proposed that CLs can stabilize the ETC complexes and store the protons in the intermembrane space^38–40^. In this study, the mRNA expression of *Plscr3* was upregulated after KLA activation, which potentially flipped CLs to the outer membrane and reduced their ability to support the ETC complexes, resulting in the loss of mitochondrial activity.

KLA-stimulated inflammation can significantly alter CL profiles. The energy production is likely to be highly regulated. We hypothesized that the changes in CL profiles during CL synthesis, remodeling, or degradation can inhibit or prevent excessive inflammation. RAW264.7 cells were supplemented with PG and CDP-DAG, which were the two reactants of CL synthesis. The incorporation of CDP-DAG into the mitochondrial membrane was not as effective as PG. To our surprise that PG supplementation caused the decrease of CL concentration, which is against our hypothesis that the increase of PG reactant will produce more CL product in the CL synthesis reaction. This indicates there are other factors involved to regulate CL synthesis. Previous research has been shown that PG is not only a reactants for CL synthesis, but may also act as a competitor to CL-binding proteins^41–45^, which also stabilized the protein complexes that require CL binding to replace the necessity of CL in mitochondria. Therefore, excessive mitochondrial PG triggered the CL hydrolysis and caused the decreases of the CL and the increases of the MLCL concentration.

On the basis of the fatty acid compositions of CLs in cells, 18:1 and 18:2 were the two most common fatty acyl chains. PG(18:1)_2_ and PG(18:2)_2_ were selected to compare the saturation effects. The incorporation of both PG types was exceptionally efficient (Fig. 3), and provides excellent models to examine mitochondria activity and elucidate the regulatory mechanisms. Moreover, several different types of CL profiles were generated, particularly in the saturation of C72 and C74 groups. The two PG types also reduced the CL concentration and increased the MLCL concentration, resulting in a 60% reduction in the CL/MLCL ratio. The mitochondrial activities between PG(18:1)_2_-supplemented and PG(18:2)_2_-supplemented cells did not exhibit significant difference. Notably, PG supplementation did not affect the mRNA expression of CL metabolism-related genes, except to inhibit Δ-6 desaturase. The mRNA expression of CL metabolism-related genes was mostly unaffected by PG supplementation; however, a slight downregulation of *Ptgs1* mRNA expression was observed. Altogether, although PG incorporation significantly altered the mitochondrial phospholipid components, the cellular mitochondrial functions remained affected.

Evidence from the present study supports the idea that PG supplementation suppresses inflammation, particularly the 358-fold reduction in *Ptgs2* mRNA expression after PG(18:1)_2_ and PG(18:2)_2_ supplementation, which was not observed in *Ptgs1* or *Alox5* mRNA expression. Although KLA activation of PG(18:1)_2_-supplemented cells did not change the CL/MLCL ratio, the ratio was significantly reduced in PG(18:2)_2_-supplemented cells. Because abnormal MLCL accumulation can trigger the aggregation of mitochondrial cristae in people with Barth syndrome^46^, PG(18:1)_2_ was a more favorable PG type for medical purposes than PG(18:2)_2_. One study reported that the *Taz* gene defects associated with Barth syndrome can reduce the *in vitro* remodeling of PG(18:2)_2_ and CL(18:2)_4_, but not PG(18:1)_2_^47^. Furthermore, the highly unsaturated forms of CLs and MLCLs can undergo oxidation during KLA-induced inflammation. Therefore, macrophage activation significantly increases CL saturation. After PG supplementation, KLA-triggered desaturation effects were ineffective, probably because desaturation was nonessential in the inflammation-suppressed conditions. KLA-induced macrophage activation drastically reduced the mitochondrial activity; however, the effects of KLA were alleviated after supplementation with both PG types. In addition, Δ-6 desaturase was inhibited after KLA addition in PG(18:2)_2_-supplemented cells.

## Materials and Methods

### Materials

Dulbecco’s modified eagle medium (DMEM) and fetal bovine serum (FBS) were purchased from Gibco (St.Louis, MO). Tetramyristoyl cardiolipin standard CL(14:0)_4_, phosphatigylglycerol standard PG(18:1)_2_, PG(18:1)_2_ and CDP-DAG(18:1)_2_ standard were purchased from Avanti Polar Lipids (Alabaster, AL). 3-deoxy-d-manno-octulosonic acid-lipid A (Kdo_2-_Lipid A; KLA), (S)-Bromoenol lactone (S-Bel), (R)- Bromoenol lactone (R-Bel) were bought from Cayman (Ann Arbor, MI). Bio-Rad protein assay, iScript^TM^ cDNA synthesis Kit and iQ™ SYBR® Green Supermix were purchased from Bio-Rad (Hercules, CA). MTT (3-(4,5-Dimethylthiazol -2-yl)-2,5-diphenyltetrazolium bromide), oligomycin and FCCP were purchased from Sigma-Aldrich (St. Louis, MO). Rotenone was acquired from Merck EMD Millipore (Billerica, MA). Mitochondria isolation kit for cultured cells was purchased from Thermo Fisher Scientific, US.

### Hydrolysis of CL by sPLA_2_

The 50 nmole CL(14:0)_4_ standard in chloroform was air-dried with N_2_ gas. The dried CL was resuspended in 498 μl of buffer (50 mM Tris-HCl, 5 mM CaCl_2_, pH 8) and sonicated for 10 times under the condition of 125 watt, 5 sec cycle, amplitude 80% to make CL vesicles. The hydrolysis reaction was initiated by adding 2 μl of purified sPLA_2_ and incubated at 40 °C water bath^28^. After 3 hours of incubation, the reaction was stopped by adding 2 ml of methanol (MeOH). Fatty acids were extracted by Bligh-Dyer methods and subjected to mass spectrometry analysis.

### Cell culture and KLA activation

Mouse leukaemic monocyte macrophage cell line (RAW264.7) was cultured in Dulbecco’s modified eagle medium with 10% fetal bovine serum, which was heat-inactivated for 30 minutes at 56 °C, 0.5% penicillin-streptomycin and 25 mM HEPES in 5% CO_2_ at 37 °C. While cells were grown to 80% confluency in culture dishes, the cells were removed from dishes by treatment with 0.05% trypsin-EDTA solutions and washed with PBS before cell seeding. For activation, the adherent RAW264.7 cells were treated with 100 ng/ml of KLA for 24 hours.

### PG and CDP-DAG Treatments

RAW264.7 cells were cultured in Dulbecco’s modified eagle medium with 10% heat-inactivated fetal bovine serum, 0.5% penicillin-streptomycin and 25 mM HEPES in 5% CO_2_ at 37 °C. The collected 6 × 10^5^ cells were transferred to a 6-cm culture dish and treated with 50 μM of CDP-DAG or PG(18:1)_2_ or PG(18:2)_2_ twice at 0 and 12 hours at 37 °C before harvest at 48 hours. In the KLA activation experiments, 100 ng/ml of KLA was added at 24 hours and harvested at 48 hours. In the multiple supplementation treatment, 50 μM of PG(18:1)_2_ were added four times to cells at 0, 12, 24 and 36 hours. The cells were then harvested at 48 hours, washed with PBS and stored in −20 °C until lipid extraction. CDP-DAG or PG(18:1)_2_ or PG(18:2)_2_ were dissolved in ethanol. The final concentration of ethanol in medium was 0.16%.

### Phospholipid Analysis

The 2 × 10^7^ RAW264.7 cells treated with PG(18:1)_2_ were harvested for mitochondria isolation following the protocol of mitochondria isolation kit (Thermo scientific, US). In brief, 800 µL of Mitochondria Isolation Reagent A. was added to cells. The samples were incubated ice for 2 minutes. Then, add 10 µL of Mitochondria Isolation Reagent B. and incubate the samples on ice for 5 minutes. After added 800 µL of Mitochondria Isolation Reagent C, the samples were gently mixed and centrifuge at 700 × g for 10 minutes at 4 °C. The samples were centrifuged at 12,000 × g for 15 minutes at 4 °C to collect the isolated mitochondria. The ingredients of the Reagent A, B and C are not disclosed by Thermo scientific. The isolated mitochondria were added 1 ml of MeOH for sonication for 60 sec. The phospholipids were extracted by Bligh-Dyer’s method. The extracted lipids were N_2_ dried and then re-suspended in 200 µL of ACN(acetonitrile)/IPA(isopropanol)/DDW(Distilled and deionized water) (65/30/5) for LC-MS/MS analysis.

### Lipid Extraction

The total lipid in the collected cells was extracted according to the Bligh-Dyer’s method as our previous experiments^48^. The internal standard, 0.2 nmole tetramyristoyl cardiolipin CL(14:0)_4_, was added to the cell pallets in 2 ml MeOH. After 3 times of pulse sonication at the condition of 125 watt, 20 sec, amplitude 80% on ice, 1 mL of dichloromethane was mixed with samples, which were further vortexed for 10 min. Then, 1 ml of dichloromethane and 1 ml of distilled deionized water were added to samples and further vortexed for 10 min. The lower phase in the glass test tube was collected by centrifugation at 2000 rpm for 5 min and the lipid sample was stored at −20 °C.

### Mass Spectrometry Analysis

The extracted total lipid was dried under nitrogen gas and immediately re-dissolved with acetonitrile/2-propanol/H_2_O (65:30:5). The samples were capped and stored in −20 °C to prevent evaporation until LC/MS Ion-Trap analysis (Bruker Corporation). Reverse phase HPLC contained solution A: ACN:H_2_O (60:40), 10 mM ammonium formate, 0.1% formic acid and solution B: IPA:ACN (90:10), 10 mM ammonium formate, 0.1% formic acid for the elution gradient from 60% solution A/40% solution B to 100% solution B in 25 min and maintained 100% solution B until 45 min. The elution was in an Acclaim RSLC 120 C18 2.1mm × 100 mm 2.2 μm column (Thermo) at a flow rate of 0.2 mL/min at 55 °C. Data were further analyzed by Bruker DataAnalysis (ver.4.1). The extract ion current (XIC) of each cardiolipin species was quantitated by their area ratio of XIC to internal standard. The total CL is the sum of all quantitated cardiolipin species. The percentage of CL was the ratio of XIC of each cardiolipin species to total XIC. Standard deviations were calculated by the STDEV function in Microsoft Excel for the error bars of the histograms and t-tests are applied to all triplicated data

### MTT Assay

RAW246.7 cells were cultured in DMEM with 10% heat inactivated fetal bovine serum in 5% CO_2_ at 37 °C. The 1 × 10^4^ cells were transferred to a 24-well culture plate and treated with 10 μM, 25 μM, 50 μM, 100 μM, 250 μM and 500 μM PG(18:1)_2_ for 24 hours at 37 °C, washed with PBS, and added medium containing 0.5 mg/ml MTT(3-(4,5-dimethyldiazol-2-yl)-2,5 diphenyl tetrazolium bromide). After 4 hours of incubation at 37 °C, MTT turned into purple formazan crystal. The medium was replaced with 200 μl of DMSO for 5 min. The absorbance of the dissolved cells in DMSO was measured at the wavelength of 550 nm.

### Seahorse Mitochondrial Assay

A total of 5 × 10^4^ RAW264.7 cells were seeded into each well of XF-24 cell culture microplate (Seahorse Bioscience) and allowed to adhere. The cells were then supplemented with 50 μM PG(18:1)_2_ or PG(18:2)_2_ twice at 0 and 12 hour. At 24 hour, 100 ng/ml KLA was added and the cells were incubated for another 24 hours. The media was replaced with DMEM pH 7.4 for 1 hour at 37 °C. The cell oxygen consumption rate (OCR) was analyzed by Seahorse XFe Extracellular Flux Analyzer (Seahorse Bioscience, Billerica, MA). The optimized concentrations of the mitochondrial inhibitors in the experiments were 10 μM oligomycin, 1 μM FCCP and 5 μM rotenone.

After seahorse mitochondrial assay, the protein concentration in each well of the XF24 cell culture microplate was measured by Bradford protein assay. The microplate was added 50 µl of 0.1% triton in PBS to each well and placed on ice for 5 min, and then 450 µl of DDW was added to each sample. The Bradford assay was started by mixing 10 µl of the prepared sample with 190 µl of 1X solution of Bradford protein-binding assay (Bio-Rad) in a 96-well plate. The absorbance at wavelength 595 nm of the plate was measured by SpectraMax M Series Multi-Mode Microplate Readers (Molecular Devices). The protein concentration was calculated based on the standard curve of BSA.

### mRNA Extraction and Reverse Transcription

RAW264.7 cells were washed with cold PBS, added 1 ml of TRIZOL reagent and then placed on ice for 5 min. The cells were transferred to a 1.5 ml eppendorf tube and added 200 μl of chloroform. The upper phase in the tube was collected after centrifugation at 12000 rpm for 15 min at 4 °C. Equal 500 μl of supernatant and isopropanol were mixed gently and kept at −20 °C for 20 min. The sample was then placed at room temperature for 10 min and collected the precipitate by centrifugation at 12000 rpm for 15 min at 4 °C. The precipitate was washed with 500 μl of ethanol and centrifuged at 9000 rpm for 5 min at 4 °C. After removing the supernatant, the sample was added 20 μl of DEPC water and heated to 50 °C for 5 min to restore the mRNA sample. The cDNA synthesis Kit from Bio-Rad was utilized for the mRNA reverse transcription. Briefly, 1 μg mRNA was mixed with 4 μl iscript reaction mix and 1 μl iscript reverse transcriptase, and then DEPC treated water was added to 20 μl of total volume. The sample was kept at room temperature for 5 min, and then heated to 42 °C for 30 min and 85 °C for 5 min. After DNA quantitation, the sample was stored at −20 °C.

### Real-time quantitative PCR

The quantitative PCR mix contains 50 ng of cDNA, 10 pmole of forward primer, 10 pmole of reverse primer and 10 μl of iQ™ SYBR® Green Supermix (Bio-Rad) in a total 20 μl volume. The sequences of the primers are provided in Supplemental Fig. S4. The samples were analyzed by MiniOpticon Real-Time PCR System (Bio-Rad, Hercules, CA). The cycling conditions were 180 sec at 95 °C for initial polymerase activation and 40 cycles of 15 sec at 95 °C and 90 sec at 60 °C. For melting curve measurement, the temperature was increased 0.1 °C per second.

## Electronic supplementary material

Supplementary Information
