# Supplementary material for: Phosphatidylglycerol Incorporates into Cardiolipin to Improve Mitochondrial Activity and Inhibits Inflammation
Source: Sci Rep. 2018 Mar 20;8:4919. doi: 10.1038/s41598-018-23190-z (PMC5861085; doi:10.1038/s41598-018-23190-z)
Supplement: Supplementary file 1 — Supplementary Information [file 41598_2018_23190_MOESM1_ESM.doc]

**Online Supplementary Material**

**Phosphatidylglycerol Incorporates into Cardiolipin to Improve Mitochondrial Activity and Inhibits Inflammation**

Wei-Wei Chen^1^, Yu-Jen Chao^1^, Wan-Hsin Chang^1^, Jui-Fen Chan^1^ and Yuan-Hao Howard Hsu^1,2^*

^1^Department of Chemistry

^2^Life Science Research Center, Tunghai University, Taichung, Taiwan

Address: No.1727, Sec 4, Taiwan Boulevard, Xitun District, Taichung 40704, Taiwan R.O.C

*Corresponding author: YHH: Phone, 011-886-4-23590121 ext-32230; FAX, 011-886-4-23590426; E-mail, howardhsu@thu.edu.tw

Supplemental Figure S1: MTT assay of PG(18:1)_2_ and CDP-DAG. (A) 10-500 μM of PG and (B) 10-250 μM of CDP-DAG were added to RAW cells in a 24-well plate for 24 hours. The cell viability was evaluated by the MTT assay.

Supplemental Figure S2: Multiple PG supplementation changes CL profile in RAW cells. The RAW cells cultured in 6-cm dish were supplemented with 50 μM of PG(18:1)_2_ in an interval of 2 days during cell passage for 8 days. The CL profile was measured by liquid chromatography- mass spectrometry.

Supplemental Figure S3: CDP-DAG supplementation does not change CL and MLCL contents. RAW cells were supplemented with 50 **μ**M of CDP-DAG twice at time 0 and 12 hours. The cells were harvested at 48 hours. The (A) CL and (B) MLCL species were analyzed by IonTrap mass spectrometry.

| **Gene ID** | **Accession Number** | **Forward Sequence 5’-3’** | **Reverse Sequence 5’-3’** | **Size**  **(bp)** | **Tm**  **(℃)** | **Intron Span** |
| --- | --- | --- | --- | --- | --- | --- |
| **Cds1** | NM_173370 | GGAGAGACGGTGGCAGATTA | CAGGTAGAGGGCGAAGGATA | 102 | 60 | yes |
| **Pgs1** | NM_133757 | ACGCTGATTGGCTCTCCTAA | CTGCTCTTGCTCCTGATGAA | 120 | 60 | yes |
| **Crls** | NM_001024385 | ACGGATTTGTTGGATGGATT | TGTAAGTGAGTGGGACTGGAA | 154 | 59 | yes |
| **Lclat1** | NM_001081071 | ACCGCCTAAGAGAAGGGAAG | TGGATGTGGGAAGAGAGTCA | 162 | 60 | yes |
| **Taz** | NM_​001173547 | CCTTATCACCGTCTCCAACC | GTCCAACGCATCAACTTCAG | 102 | 59 | yes |
| **Fads2** | NM_019699 | CAAAACCAACCACCTGTTCTT | AAAGGCTGTGACGAGGGTAG | 121 | 59 | no |
| **Pla2g6** | NM_001199023 | GAGACTGCCTTCCATTACGC | TCAGCCCTTGGTTGTTTACC | 106 | 60 | yes |
| **Pnpla8** | NM_26164 | TTCCTTTCTCGTCCCACTGA | GCAGACACTTCCTGTTCTTCG | 116 | 61 | no |
| **Pld6** | NM_001290283 | CTCTGCCTCTTCGCCTTCT | ACCTGTATCCCTGCCTTGC | 152 | 60 | yes |
| **Cycs** | NM_007808 | AAATCTCCACGGTCTGTTCG | TGCCCTTTCTCCCTTCTTCT | 187 | 60 | yes |
| **Bid** | NM_007544 | AGCCCTTGATGAGGTGAAGA | GCAAAGATGGTGCGTGACT | 113 | 60 | no |
| **Plscr3** | NM_001168497 | CTGGACTTGTGGCTGTGGTA | GGCATCTGTGAGGGCTTCT | 121 | 60 | yes |
| **Ptgs1** | NM_008969 | ACAGTGCGGTCCAACCTTAT | AGAGGGCAGAATGCGAGTAT | 111 | 59 | yes |
| **Ptgs2** | NM_011198 | CCCCCACAGTCAAAGACACT | ATCATCAGACCAGGCACCA | 112 | 60 | no |
| **Alox5** | NM_009662 | CATCAAGAGCAGGGAGAAGC | CATAGTTGGAGGAGCGTTGG | 130 | 61 | yes |
| **Gapdh** | NM_008084 | AACTTTGGCATTGTGGAAGG | GGATGCAGGGATGATGTTCT | 132 | 65 | yes |

Supplemental Figure S4: Sequences of the primers utilized in RT-qPCR
